# Association between dietary behavior and puberty in girls

**DOI:** 10.1186/s12887-024-04840-w

**Published:** 2024-05-21

**Authors:** Yiming Du, Wu Yan, Francis Manyori Bigambo, Qiaoli Zhou, Chenguang Ma, Wei Gu, Xu Wang

**Affiliations:** 1 Department of Pediatrics, Zhongda Hospital, Southeast University, Nanjing, 210009 China; 2 Department of Endocrinology, Children’s Hospital of Nanjing Medical University, 72 Guangzhou Rd, Nanjing, 210008 China; 3 Department of Children Health Care, Children’s Hospital of Nanjing Medical University, Nanjing, 210008 China

**Keywords:** Dietary behavior, Onset of puberty, Precocious puberty, Girls, Children

## Abstract

**Introduction:**

Over the decades the trends of early onset of puberty have been observed in children, particularly in girls. Research evidence has reported diet to be among the most important risk factors for puberty onset. This study evaluated the association between dietary behavior and puberty in girls.

**Methods:**

We enrolled 201 girls with the main complaints of breast development as the cases at the Endocrine Department of Nanjing Children's Hospital. The cases were divided into breast development with central priming and breast development without central priming groups and were matched with 223 normal health girls with no breast development (control group). We used the modified Child Eating Behavior Questionnaire (CEBQ) to conduct a face-to-face interview about dietary behavior. Sample t-test or Mann Whitney U test or Chi-square test, the analysis of variance or Kruskal Wallis test, and least significant difference (LSD) were used to compare differences between the groups, Bonferroni was used to correct the *p*-value, and logistic regression was used to analyze risk factors for puberty onset.

**Results:**

A total of 424 girls participated in this study, among them, 136 were cases with breast development with central priming, 65 were cases with breast development without central priming, and 223 were normal health girls with no breast development. Age of the participants ranged from 4.5 to 9.3 years. There were significant differences in food response (*p* < 0.001), dietary restriction (*p* < 0.001), frequencies of vegetable intake (χ^2^ = 8.856, *p* = 0.012), drinking milk (χ^2^ = 23.099, *p* = 0.001), and borderline statistical difference in a total score of unhealthy dietary behavior (*p* = 0.053) among the cases and controls. However, in the post hoc analysis, these dietary behaviors were significant differences between the girls with breast development with central priming and the control groups. Moreover, girls in the breast development with central priming group had significantly higher bone age (BA), uterine body length, ovarian volume, basal luteinizing hormone (LH), basal follicle-stimulating hormone (FSH), peak LH, peak FSH, estradiol (E2), and free triiodothyronine (FT3) compared to those in the breast development without central priming group. In the multivariate logistic regression, only uterine body length was associated with increased risk of breast development with central priming (OR = 1.516, 95%CI: 1.243–1.850).

**Conclusion:**

There were significant differences in dietary behaviors among girls with breast development with central priming and normal health girls with no breast development, and uterine body length was associated with an increasing risk of breast development with central priming among girls with breast development.

## Introduction

Adolescence is the transitional period from childhood development to adulthood. The indicators of puberty development include the appearance of secondary sexual characteristics, gonadal enlargement, rise in serum gonadotropin and sexual hormone levels, and linear growth acceleration. Precocious puberty refers to the development of secondary sex characteristics before 7.5 years old or menarche onset before 10.0 years old in girls, and the development of secondary sex characteristics before 9.0 years old in boys [1]. Premature youth development not only affects year-end height [2], early menarche, and inconvenience in life, but also causes psychological problems in children, and even increases the risk of hormone related cancer [3].

Over the past 30 years, there has been a significant trend of early onset of puberty in children in China, and the number of children with precocious puberty has significantly increased. A study based on the school population showed that the prevalence of Tanner stage precocious puberty was 6.29%, with girls having a higher prevalence rate (14.23%) than boys (1.54%) [4], but the specific reason is unknown. Numerous studies have shown that diet is one of the most important factors affecting the duration of youth development [3]. Diet to a certain extent controls the time of youth development and promotes physiological changes related to the initiation of youth [5].

Dietary behavior is an important component of children's diet. With the improvement of living standards and the impact of the dietary environment, children's unhealthy dietary behavior is becoming increasingly serious [6]. A previous study has shown that poor dietary behavior may be related to the onset of puberty in children [7], but the number of related studies is relatively small. To further understand the relationship between dietary behavior and adolescent onset, we conducted a case–control study to explore the impact of dietary behavior on adolescent onset in girls, the results can be helpful to improve the prevention strategy of female precocious puberty, thus reducing the chance of girls' precocious puberty.

## Methods

### Study design, population, and sample size

In this study, the cases were girls who reported "breast development" as the main complaint at the Endocrinology Department of Nanjing Children's Hospital from January 2022 to January 2023 and were matched with healthy girls without breast development and other diseases based on age. The sample size was computed using the formula [8]:

1 $$\text{Sample size = }\frac{r+1}r\frac{\left(Z\beta+Z\alpha/2\right)^2\text{P}\left(1-P\right)}{\left(P1-P2\right)^2}$$

Whereby r = cases to control ratio = 1, for an equal number of cases and controls. Z_α/2_ = Standard normal variate of significance 0.05/2 = 1.96 and Z_β_ = Standard normal variate of power = 90% = 1.28. P1 = Proportion in cases = The proportion of tanner staging precocious puberty = 0.14 for girls as reported elsewhere [4]. P2 = Proportion in control = The proportion of normal healthy girls (without precocious puberty) was assumed to be 0.27 after the author's thorough discussion with a statistician. P = Average proportion between cases and controls = P1 + P 2. After computation, the sample size obtained was 201 pairs. To have enough age representation when matching the cases and controls and for the effectiveness of the sample size, we recruited 201 cases and 223 controls. The inclusion criteria for the breast development group (cases) include: 1) Girls aged between 4.5 and 9.3 years old whose breast development is Tanner II or above [9]. 2) The guardians of the children involved signed informed consent. 3) Children were Nanjing residents. The exclusion criteria were children with secondary central precocious puberty, such as central nervous system space occupying, infection, trauma, postoperative, radiotherapy or chemotherapy, congenital dysplasia, and other primary diseases that may lead to breast development, such as Congenital adrenal hyperplasia, McCune Albright syndrome, and congenital hypothyroidism. The control group was normal healthy girls with no breast development. This study was approved by the Ethics Committee of the Children's Hospital affiliated with Nanjing Medical University (202101014–1), and the guardians of the children involved signed the informed consent.

### Questionnaire information collection

General information about girls collected includes date of birth, height, and weight. According to literature and clinical practice [10], we used the modified Child Eating Behavior Questionnaire (CEBQ) to conduct a face-to-face interview. Trained medical personnel guided children’s guardians in scanning and filling out the questionnaire. The content of the questionnaire was about girls' eating behavior within three months before breast development with the following 9 items: picky eating, food response, bad eating habits, satiety response, exogenous eating, emotional eating, food preference, diet restriction, and junk food craving. A 5-point classification method was used to represent frequency, which is never/rarely/sometimes/mostly/always, and assign a score of 1–5 points in sequence. The higher the score, the more dietary behavior problems children have.

Moreover, we collected the dietary preferences and intake of girls within the first three months of breast development, including the frequency of consumption of meat, fruits, soy products, starch, eggs, and milk. Children’s guardians were asked to select at least one type of food (meat, fruits, soy products, starch, and eggs) their children eat the most within the first three months of breast development. The responses were recorded as frequency (percentage). Also, children’s guardians were asked how often their children drink milk within the first three months of breast development. A 4-point classification method was used to represent frequency including every day, more than 3 days per week, less than 3 days per week (including 3 days), and not drinking.

We computed the questionnaire (modified CEBQ) reliability and validity tests (KMO value = 0.860, sphericity test *p* < 0.001). The Cronbach's α coefficient is 0.869, indicating that the questionnaire has good structural validity.

### Assessment of puberty development

Trained medical personnel carried out on-site assessments and evaluations to obtain data on the development of secondary sex characteristics in girls. The girls' breast development was assessed and evaluated using visual and palpation methods according to Tanner's staging as reported in the expert consensus (2022) on the diagnosis and treatment of central precocious puberty (CPP) [1]. In our study girls with breast development reaching Tanner II or above were divided into two groups as follows: Girls with breast development reaching Tanner II or above and gonadotropin-releasing hormone (GnRH) stimulation test results [11] indicating gonadal axis activation [1] were identified as the breast development with central priming group (136 cases) and those with breast development reaching Tanner II or above, but no indication of gonadal axis activation in the GnRH stimulation test were identified as breast development without central priming group (65 cases). Simultaneously, 223 healthy girls with no breast development were selected as the control group.

### Laboratory examination

For girls with breast development, fasting blood specimens were collected during the morning for the evaluation of the hormones including luteinizing hormone (LH), follicle-stimulating hormone (FSH) baseline values, and peak values after GnRH stimulation test, estradiol (E2), thyroid function [thyroid stimulating hormone (TSH), free triiodothyronine (FT3), free thyroxine (FT4)], which were analyzed by electrochemiluminescence immunoassay (Roche Diagnostics GmbH, Mannheim, Germany). Bone age (BA) was measured by a hand and wrist x-ray, while the uterine length, ovarian length, width, and thickness were measured by ultrasound with a bladder full, and BMI (2), bone age index (BAI (3), bone age difference (4) and the volume of the left and right ovaries (5). CA refers to chronologic age. Take the larger value of the ovarian volume on both sides as the ovarian volume. In addition, a bone age difference of ± 1 indicates normal BA and ≥ 1 indicates advanced BA.

2 $$BMI=\frac{weight}{height^2}\left(\frac{kg}{m^2}\right)$$

3 $$\text{BAI = }\frac{BA}{CA}$$

4 $$\text{Bone age difference = BA - CA }\left(years\right)\text{ }$$

5 $$\mathrm{Ovarian}\;\mathrm{volume}\;=\;\mathrm{long}\;\mathrm{diamater}\;\times\;\mathrm{Wide}\;\mathrm{diameter}\;\times\;\mathrm{thickness}\;\times\;0.5233\;\left(\mathrm{ml}\right)$$

### Statistical analysis

Data analysis was conducted using SPSS 26.0 (IBM, Armonk, New York, USA). For parametric data, the continuous variables were represented as mean ± standard deviation, and an independent sample t-test was used for comparison of the difference between the two groups. The analysis of variance was used for comparison of differences between multiple groups. Then, the least significant difference (LSD) method was used for pairwise comparison if there were differences between groups. But for non-parametric data, continuous variables were presented as the median and interquartile range (IQR), and the Kruskal Wallis test was used to compare the differences between multiple groups, if there were significant differences between the groups, the Mann Whitney U test was used for pairwise comparison. The categorical variables were expressed as frequency (%), and the Chi-square test was used to compare differences between the groups. Bonferroni was used to correct the *p-*value when comparing pairs with differences between the groups. The data normality test was checked by Shapiro–Wilk. The multivariate logistic regression model was performed for the characteristics that showed significant differences between breast development with central priming and breast development without central priming groups. The test level was set at both sides α = 0. 05, *p* < 0.05 was considered statistically significant. 

## Results

A total of 424 girls ages ranging from 4.5 to 9.3 years participated in this study, including 201 in the case groups (136 in the breast development with central priming group and 65 in the breast development without central priming group), and 223 in the control group. General information of the participants showed that age (F = 3.681, *p* = 0.026), height (F = 4.935, *p* = 0.008), body weight (F = 4.972, *p* = 0.007), and BMI (F = 10.934, *p* < 0.001) were significant differences between the case groups (breast development with central priming group and breast development without central priming group) and the control group (no breast development) (Table 1).

**Table 1:** General information about the study participants (*N*=424)

	**Breast development with central priming group (*N*=136)**	**Breast development without central priming group (*N*=65)**	**Control group (*N*=223)**	**F**	***P* value**
Age (years)	7.94±1.24_a_	7.47±1.06_b_	7.77±1.11_a, b_	3.681	0.026
Height (cm)	132.15±8.30_a_	129.32±8.20_b_	129.20±9.46_b_	4.935	0.008
Body weight (kg)	28.93±5.15_b_	27.73±5.62_b_	31.30±11.77_a_	4.972	0.007
BMI (kg/m²)	16.50±1.96_b_	16.50±2.43_b_	18.55±5.82_a_	10.934	<0.001

After analysis of variance, if there were differences between the three groups, the LSD method was used for pairwise comparison. Letters are used to indicate the comparison results between the groups. The same letter indicates that the difference is not statistically significant, while different letters indicate that the difference is statistically significant, a>b. Significant at *p*< 0.05

**Fig. 1:**
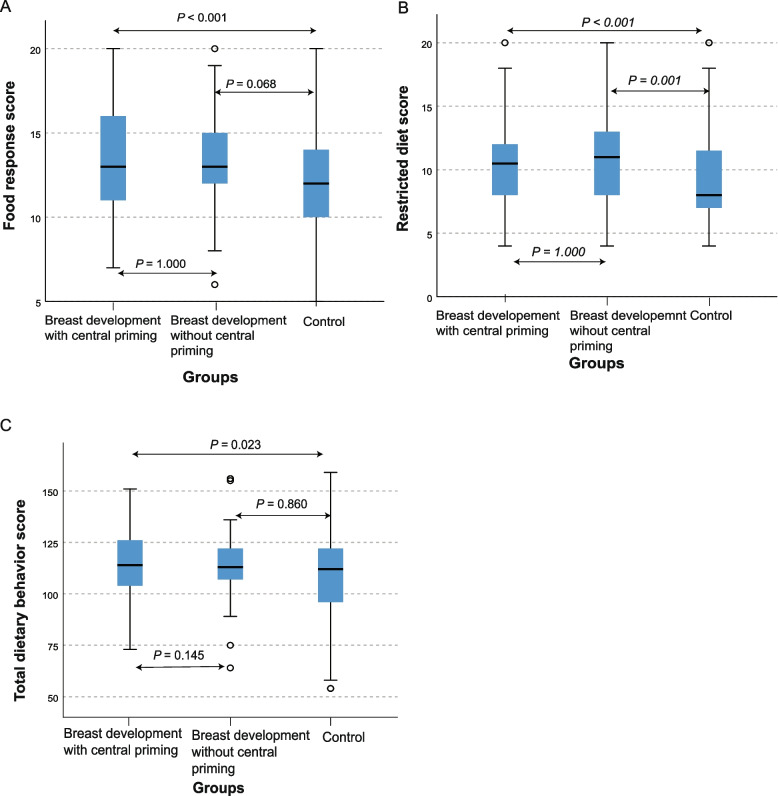
Box plots for the dietary behavior habits of the three groups of girls including those with breast development with central priming (*N* = 136), breast development without central priming (*N* = 65), and the control (normal health girls with no breast development (*N* = 223))

In the dietary behavior, there were significant differences in food response (*p* < 0.001) and restricted diet (*p* < 0.001), and the total score of dietary behavior showed a borderline statistical difference (*p* = 0.053) in the three groups of girls (the breast development with central priming group, the breast development without central priming group, and the control group) when comparing the groups using the Kruskal Wallis test. On the other hand, we did not find significant differences in picky eating, food preference, junk food cravings, bad eating habits, overeating, exogenous eating, and emotional eating in the three groups of girls (Table 2). Moreover, we performed a post hoc analysis using the Mann Whitney U test for the pairwise comparison and found there were significant differences in food response, restricted diet, and total score of dietary behavior when comparing girls with breast development with central priming and the control groups. However, these significant differences were not observed when comparing the case groups only (breast development with central priming and breast development without central priming groups) (Fig. 1A-C), indicating that the differences seem not to be related to the presence or absence of central priming development.

**Table 2:** Score of girls' eating behavior among the three groups

	**Breast development with central priming group (*N*=136)**	**Breast development without central priming group (*N*=65)**	**Control group (*N*=223)**	***P* value**
	Median (IQR)	Median (IQR)	Median (IQR)	
Picky eaters	11.00 (9.00-14.00)	12.00 (9.00-14.00)	11.00(8.00-13.00)	0.118
Food preferences	8.00(7.00-9.00)	8.00(6.50-9.00)	8.00(7.00-9.00)	0.401
Junk food craving	20.50 (16.25-24.00)	20.00 (17.00-23.50)	20.00 (16.00-22.00)	0.285
Poor eating habits	9.00(7.00-11.00)	9.00 (7.00-10.50)	9.00 (7.00-11.00)	0.997
Satiety response	0.00 (-2.00- 3.00)	0.00 (-2.00-2.50)	0.00 (-2.00-2.00)	0.987
Exogenous feeding	14.50 (11.00-17.00)	13.00 (11.50-15.50)	14.00 (11.00-16.00)	0.155
Emotional eating	10.00(5.25-10.00)	10.00 (6.00-11.00)	10.00 (5.00-11.00)	0.143
Food response	13.00 (11.00-16.00)^a^	13.00 (11.50-15.00)^a^^, b^	12.00 (10.00-14.00)^b^	**<0.001**
Restricted diet	10.50 (8.00-12.00)^a^	11.00 (8.00-13.00)^a^	8.00 (7.00-12.00)^b^	**<0.01**
Total score of dietary behavior	114.00 (104.00-126.00)^a^	113.00 (107.00-122.00)^a, b^	112.00 (96.00-122.00)^b^	**0.053**

IQR, Interquartile range. Kruskal Wallis test was performed to compare the differences between the three groups, if there were significant differences, the Mann Whitney U test was used for pairwise comparison. Letters are used to indicate the comparison results between the groups. The same letter indicates that the difference is not statistically significant, while different letters indicate that the difference is statistically significant, a>b. Significant at *p* <0.05

In terms of dietary preferences and intake of girls, we observed significant differences in frequencies of vegetable intake (χ2 = 8.856, *p* = 0.012) and drinking milk (χ2 = 23.099, *p* = 0.001) among the three groups of girls. There were no significant differences in the frequencies of intake of meat, fruits, soy products, starch, and eggs among the three groups of girls (Table 3).

**Table 3:** Dietary preference and intake of the three groups of girls

	**Breast development with central priming group (*N*=136)**	**Breast development without central priming group (*N*=65)**	**Control group (*N*=223)**	***χ***^**2**^	***P* value**
Types of foods consumed frequently					
Meat (%)	113 (83.1)	53(81.5)	187 (83.9)	0.198	0.906
Vegetables (%)	72 (52.9)_b_	33 (50.8)_a, b_	148 (66.4)_a_	8.856	0.012
Fruits (%)	94 (69.1)	47 (72.3)	174 (78.0)	3.669	0.160
Bean products (%)	44 (32.4)	22 (33.8)	90 (40.4)	2.615	0.271
Starch (rice, noodles, coarse grains, etc.) (%)	76 (55.9)	34 (52.3)	130 (58.3)	0.777	0.678
Eggs (%)	72 (52.9)	38 (58.5)	131 (58.7)	1.242	0.537
Frequency of drinking milk				23.099	0.001
Every day (%)	84 (61.8)_a_	48 (73.8)_a_	101 (45.3)_b_		
More than 3 days per week (%)	31 (22.8)_a_	9 (13.8)_a_	60 (26.9)_a_		
3 days or less per week	17 (12.5)_a_	6 (9.2)_a_	42 (18.8)_a_		
Not drinking (%)	4 (2.9)_a_	2 (3.1)_a_	20 (9.0)_a_		

Bonferroni was used to correct the *p* value when comparing two pairs with differences in the Chi-square test among the three groups, and the alphabet was used to indicate the comparison results between the groups. The same letter indicates that the difference is not statistically significant, and different letters indicate that the difference is statistically significant, a>b. Significant at* p*< 0.05

The laboratory examination results of girls in the case groups showed that in the Mann Whitney U test, there were significant differences in basal LH ( *p* < 0.001), basal FSH ( *p* < 0.001), peak LH ( *p* < 0.001), peak FSH (*p* = 0.015), E2 ( *p* < 0.001), FT3 (*p* = 0.009), BA ( *p* < 0.001), uterine length ( *p* < 0.001), and ovarian volume (*p* = 0.005) between the breast development with central priming and breast development without central priming groups (Table 4).

**Table 4:** Laboratory examination between breast development with central priming and without the central priming groups

	**Breast development with central priming group (*N*=136)**	**Breast development without central priming group (*N*=65)**	***P* value**
	**Median (IQR)**	**Median (IQR)**	
BA (years)	10.00 (9.00-11.00)	10.00 (8.80-10.50)	<0.001
BAI	1.17 (1.11-1.33)	1.32 (1.21-1.37)	0.351
Uterine body length (mm)	24.00 (22.00-27.00)	21.00 (20.00-21.00)	<0.001
Ovarian volume (ml)	1.59 (1.15-1.93)	0.77 (0.56-1.63)	0.005
Basal LH (mIU/ml)	0.62 (0.31-1.030	0.20 (0.15-0.29	<0.001
Basal FSH (mIU/ml)	3.98 (2.93-4.97)	2.44 (1.58-3.55)	<0.001
Peak LH (mIU/ml)	14.36 (9.52-23.10)	3.70 (2.81-4.37)	<0.001
Peak FSH (mIU/ml)	12.21 (10.15-14.54)	10.64 (8.07-12.74)	0.015
E2 (pmol/L)	68.00 (18.35-122.80)	21.48 (18.35-63.87)	<0.001
TSH (mIU/ml)	2.73 (1.98-3.63)	2.58 (1.57-3.80)	0.192
FT3 (pmol/L)	6.97 (6.36-7.64)	6.78 (6.65-7.11)	0.009
FT4 (pmol/L)	18.12 (16.86-19.06)	17.46 (17.01-17.87)	0.781

The Mann Whitney U test was used to compare the differences between the two groups

*IQR* Interquartile range, *BA* Bone age, *BAI* Bone age index, *LH* Luteinizing hormone, *FSH* Follicle-stimulating hormone, *E2* Estradiol, *TSH* Thyroid stimulating hormone, *FT3* Free triiodothyronine, *FT4* Free thyroxine

Significant at *p< *0.05

Moreover, we performed multivariate logistic regression analysis for the characteristics that showed significant differences between breast development with central priming and breast development without central priming groups except for the peak LH and peak FSH because some peak LH and peak FSH values are described as specific numbers while others are recorded as greater than a certain value, therefore, to ensure the robustness and interpretability of the regression results, the peak LH and peak FSH were excluded. The results showed that an increase in uterine body length was a risk factor for breast development with central priming. Specifically, for every 1 mm increase in uterine length, the risk of breast development with central priming increases by 1.516 times (95%CI: 1.243–1.850). Furthermore, we find the marginal results for the associations of basal FSH and LH with breast development with central priming (Table 5), indicating no significant associations.

**Table 5:** Multivariate logistic regression analysis of breast development with and without central priming groups

	**Coefficient**	**Error**	**Wald**	**OR value**	**95%CI**	***P*value**
BMI (kg/m²)	-0.069	0.100	0.477	0.934	0.768-1.135	0.490
Age difference in bone age (years)	0.046	0.173	0.069	1.047	0.745-1.470	0.792
Uterine body length (mm)	0.416	0.101	16.82	1.516	1.243-1.850	<0.001
Ovarian volume (ml)	0.068	0.303	0.050	1.070	0.591-1.940	0.823
Basal LH (mIU/ml)	2.111	1.128	3.505	8.259	0.906-73.315	0.061
Basal FSH (mIU/ml)	0.395	0.228	2.994	1.484	0.949-2.322	0.084
E2 (pmol/L)	0.011	0.007	2.316	1.011	0.997-1.026	0.128

*BMI* Body mass index, *LH* Luteinizing hormone, *FSH* Follicle-stimulating hormone, *E2* Estradiol, *OR* Odds ratio, *CI* Confidence interval

Significant at *p*< 0.05

## Discussion

In this matched case–control study, we explored the impact of dietary behavior on puberty in girls. The results showed that girls in the breast development groups (cases) had higher responses to overeating, more restrictive dietary behaviors, and overall poor dietary habits compared to the control group. In terms of dietary preference and intake, girls in the control group consumed vegetables more frequently than girls in the breast development groups. The behavior of drinking milk every day was significantly higher in the breast development groups than in the control group. Furthermore, girls in the breast development with central priming group had greater basal LH, basal FSH, peak LH, peak FSH, E2, FT3, BA, uterine body length, and ovarian volume compared to those in the breast development without central priming group. However, only uterine body length was a risk factor for breast development with central priming in a multivariable logistic regression.

In this study, we found that the total score of dietary behavior of girls in the breast development groups was higher than those in the control group. The obesity susceptibility test conducted by CarnellS et al. [12] observed that as early as the age of 3 years, satiety and food orientation are associated with obesity; and by school age, low satiety responsiveness and high food orientation promote weight gain. Parkinson et al. also observed similar results [13]. In addition, Braet et al. [14] used the Dutch Eating Behavior Scale, and Stunkard et al. [15] used the three-factor eating behavior scale as a measuring tool to confirm that the food response dimension had a strong positive correlation with BMI. The above research indicates that the higher the response of children to food, the higher the risk of obesity, and obesity is one of the important risk factors for precocious puberty [16]. Therefore, we believe that more food orientation behavior is an important risk factor for breast development and gonadal axis activation in girls. Even though in our study, insufficient evidence was observed for the association between BMI and breast development with central priming, despite the significant difference in BMI found between the case groups (breast development with central priming group and breast development without central priming group) and the control group (no breast development). Studies discrepancies may be caused by various reasons including variations in study designs, sample sizes, populations, and unmeasured confounders.

In the case of dietary restriction, we found that all three groups of girls had varying degrees of dietary awareness, but overall, girls in the breast development groups exhibited more dietary restriction behaviors than girls in the control group. We speculate that dietary restriction behavior may be related to the existence of weight related psychological stress in children, but the specific relationship between dietary restriction behavior, psychological stress, and adolescent onset in girls needs further research.

Overall, compared to the control group, the girls in the breast development groups had poorer dietary behavior. Therefore, we believe that overall poor dietary behavior is a risk factor for breast development, but it is not clear whether it is accompanied by gonadal axis activation. Moreover, we found that compared to girls in the breast development groups, girls in the control group consumed vegetables more frequently and milk less frequently every day. The above results are consistent with previous studies [17, 18], which found that plant protein is a protective factor for youth development, while excessive milk intake (such as a daily intake of 34 g or more) is a risk factor for youth development.

In the laboratory test results, we found that there were differences in bone age (BA) among girls in the breast development groups, and all groups indicated that BA was advanced, but there was no significant difference in BAI. There is a large body of literature regarding BAI as an indicator of bone maturity, and it is believed that BAI can more accurately reflect the degree of BA changes in different individuals compared to simple BA and bone age differences [19]. However, the consensus of CPP diagnosis and treatment experts (2022) does not mention this indicator and regards BA as one of the diagnostic criteria for CPP. Therefore, we believe that children's dietary behavior is not related to BA.

In the consensus of CPP diagnosis and treatment experts (2022), the results of pelvic ultrasound examination in girls showed a uterine length of 3.4–4.0 cm, an ovarian volume of 1-3 ml, and multiple follicles with a diameter of ≥ 4 mm as signals of puberty initiation. Although uterine body length was associated with an increased risk of breast development with central priming, according to the above criteria, the length of the uterine body of both groups of girls did not meet the criteria for puberty initiation, but the ovarian volume met the criteria for puberty initiation. It is worth noting that the length of the uterus in the expert consensus does not specifically refer to the length of the uterine body, but may also refer to the sum of the length of the uterine body and the length of the cervix. In this study, the results of pelvic ultrasound examinations in girls were all based on the length of the uterine body, which was measured by experienced ultrasound physicians after the girls had fully held their urine. The ovarian volume was calculated based on the formula in expert consensus, but not all girls have multiple enlarged follicles visible on ultrasound examination.

The advantages of this study include: We divided the case groups and control group using Tanner staging, abandoning the age indicator of precocious puberty in girls and avoiding misdiagnosis caused by memory bias. We further grouped girls in the case groups according to whether breast development is accompanied by central priming or not and further explored the relationship between children's dietary behavior and puberty.

This study also has some limitations: Firstly, our research population is limited to a small number of girls seeking medical treatment at Nanjing Children's Hospital and the population is relatively one center. Secondly, most of the information in this study including the general information, dietary habits, and dietary preference and intake of girls were obtained from girls and their guardians through a questionnaire, and there may be a recall bias. In addition, the recall time for collecting dietary behaviors was three months before or after breast development, this could lead to bias. Thirdly, breast development for some children may have occurred more than a year ago, this could also increase the risk of recall bias. Fourthly, dietary preferences and intake may vary due to seasonal differences. In this study, for the dietary habits of children, the frequency of each subcategory indicator is represented by the 5-class classification method, but there is no specific definition of the specific frequency for each classification.

## Conclusion

In summary, this study investigated the association between dietary behavior and puberty in girls. We found that there were significant differences in dietary behaviors among girls with breast development and normal health girls with no breast development, and uterine body length was associated with an increasing risk of breast development with central priming among girls with breast development. The impact of children's dietary behavior on puberty is manifested as a long-term and multifactorial comprehensive effect. Therefore, parents and society should attach importance to the cultivation of good dietary habits in children, and parents should actively learn about child feeding knowledge to promote healthy growth of children and minimize the occurrence of precocious puberty.

## Data Availability

The datasets analyzed during this study are available from the corresponding author upon reasonable request.
